# A chromosome-scale draft genome sequence of horsegram (*Macrotyloma uniflorum*)

**DOI:** 10.46471/gigabyte.30

**Published:** 2021-10-08

**Authors:** Kenta Shirasawa, Rakesh Chahota, Hideki Hirakawa, Soichiro Nagano, Hideki Nagasaki, Tilak Sharma, Sachiko Isobe

**Affiliations:** ^1^ Kazusa DNA Research Institute, 2-6-7 Kazusa-kamatari, Kisarazu, Chiba 292-0818, Japan; ^2^ Department of Agricultural Biotechnology, CSK Himachal Pradesh Agricultural University, Palampur, Himachal Pradesh 176062, India; ^3^ ICAR – Indian Institute of Agricultural Biotechnology, Ranchi, Jharkhand 834010, India; ^4^ Forest Tree Breeding Center, Forestry and Forest Products Research Institute, 3809-1 Ishi, Juo, Hitachi, Ibaraki 319-1301, Japan

## Abstract

Horsegram (*Macrotyloma uniflorum* [Lam.] Verdc.) is an underutilized warm-season diploid legume (2*n* = 20, 22). Because of its ability to grow under water-deficient and marginal soil conditions, horsegram is a preferred choice in the era of global climate change. In recognition of its potential as a crop species, we generated and analyzed a draft genome sequence for a horsegram variety, HPK-4. Ten chromosome-scale pseudomolecules were created by aligning Illumina scaffold sequences onto a linkage map. The total length of the ten pseudomolecules was 259.2 Mbp, covering 89% of the total length of the assembled sequences. A total of 36,105 genes were predicted on the assembled sequences. Diversity analysis of 89 horsegram accessions by dd-RAD-Seq identified 277 single nucleotide polymorphisms (SNPs), suggesting narrow genetic diversity among the horsegram accessions. This is the first attempt to generate a draft genome sequence of horsegram and will provide a reference for sequence-based analysis of horsegram germplasm.

## Data description

### Background

Horsegram (*Macrotyloma uniflorum* [Lam.] Verdc.) (NCBI:txid271171), is an underutilized warm-season diploid legume (2*n* = 20, 22). It belongs to the Fabaceae family of the Phaseoleae tribe, and is cultivated mainly in semi-arid regions of the world. On the Indian subcontinent, horsegram is consumed primarily as a food legume, whereas in Africa and Australia it is grown mainly for use as a concentrated animal feed and fodder. This self-pollinating plant is thought to have originated in Africa because most of its 32 wild species exist there [1], and the Northwestern Himalayan region is considered its secondary center of origin [2]. Horsegram may have been domesticated as *M. uniflorum* var. uniflorum in the southern part of India, but its probable progenitor, *M. axillare*, has not been reported in India. Therefore, the process by which cultivated horsegram was domesticated from its wild ancestors has not yet been established [3].

Because of its ability to grow under water-deficient and marginal soil conditions, horsegram is a preferred choice in the era of global climate change. Horsegram contains 16.0–30.4% protein [4], and constitutes an important source of dietary protein for the undernourished population in south Asia. In addition, the seeds are a rich source of lysine and vitamins [5], and its antioxidant, antimicrobial, and unique antilithiatic properties make it a food of nutraceutical importance [6–8]. As a result of horsegram’s medicinal importance and ability to thrive under drought-like conditions, the US National Academy of Sciences has identified this legume as a potential food source for the future [9].

### Context

The existence of many wild and unsolicited characteristics makes horsegram a less favorable legume for commercial cultivation, although it does possess numerous attributes that make it a potential food legume for warm arid regions. In addition, there is a lack of genetic and molecular tools with which to genetically enhance horsegram. To elucidate the potential of this food legume species, we generated and analyzed a draft genome sequence for HPK-4, a horsegram cultivar commercially released by CSK Himachal Pradesh Agricultural University (HPAU), Palampur, India. This variety, which has dark-brown seeds, is under cultivation in many parts of the Northwestern Indian Himalayan region. It is resistant to anthracnose (*Colletotrichum truncatum*) and tolerant to abiotic stresses such as drought, salinity, and heavy metals. This is the first attempt to generate a draft genome sequence of this ‘orphan’, but it is an important food legume species and will provide a reference for sequence-based analysis of horsegram germplasm to elucidate the genetic bases of important traits.

## Methods

### Whole genome sequencing and assembly of horsegram

**Figure 1.**
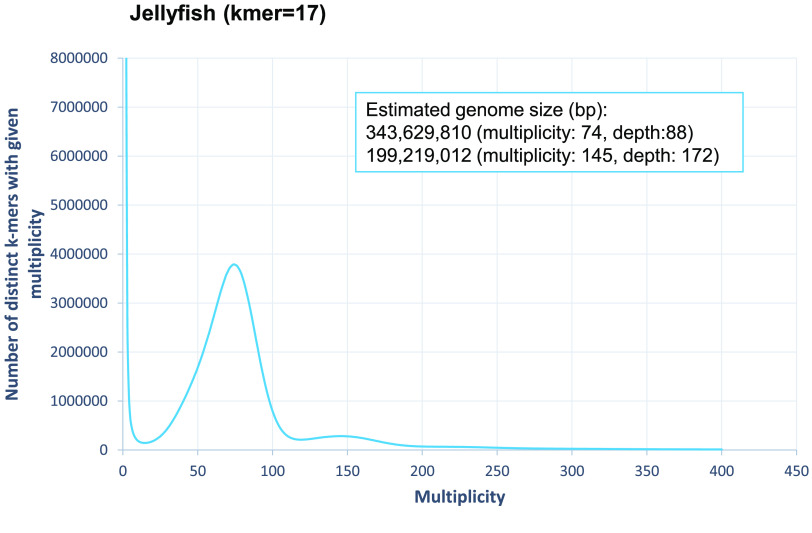
Genome size estimation using Jellyfish with the distribution of the number of distinct *k*-mers (*k* = 17) with the given multiplicity values.

The genome sequences of a horsegram variety, HPK-4, bred at CSK-HPAU, were generated from a paired-end (PE) library by Illumina HiSeq 2000 with a total length of 37.9 Gbp (gigabase pairs) [10]. All data analysis for this study was performed on Linux servers running Red Hat Enterprise Linux Server 7.1. Using the Jellyfish v1.1.6 program (RRID: SCR_005491) [11], the genome size of HPK-4 was estimated to be approximately 343.6 Mbp (megabase pairs) (Figure 1). The parameters used in the analysis are listed in Table 1.

**Table 1:** Parameters used in each program.

Program name	Parameters or BUSCO data set	Comments
Jellyfish v1.1.6	–m 17 –s 1000000000 –t 32 –C	
SOAPdenovo2 r223	–K (61 71 81 91) –R –F –p 8	
SSPACE v2.0	–x 0 –z 0 –k 3 –a 0.7 –n 15 –T 8 –g 0 –v 1	
GapFiller v1.10	–m 30 –o 5 –r 0.7 –n 10 –d 50 –t 10 –g 0 –T 8 –i 1	
Platanus v1.2.1	–t 12 –m 300	
MaSuRCA v2.3.2	Default Parameters	
TruSPAdes v3.6.2	Default Parameters	
RepeatMasker v3.2.9	–poly –x –lib	
RepeatScout v1.0.5	Default Parameters	
BRAKER1 v1.9	Default Parameters	
BUSCO v3.0	Embryophyta, odb10	
Samtools 0.1.19	samtools mpileup mpileup –d 10000000 –D –u	
bcftools 0.1.19	bcftools view –c –g –v	
vctools 0.1.12b	vcftools_0.1.12b/bin/vcftools –remove-indels –min-alleles 2 –max-alleles 2 –minDP 5 –minQ 214 –max-missing 1	SNP filterfing for 8 F_2_ WGS
vctools 0.1.12b	vcftools_0.1.12b/bin/vcftools –remove-indels –min-alleles 2 –max-alleles 2 –minDP 10 –minQ 50 –max-missing 0.2	SNP filterfing for 214 F_2_TAS
vctools 0.1.12b	vcftools_0.1.12b/bin/vcftools –remove-indels –min-alleles 2 –max-alleles 2 –minDP 5 –minQ 999 –max-missing 0.5 –maf 0.05	SNP filtering for 89 population
JoinMap 4	Kosambi’s mapping function, linkage with rec. frec. Smaller than 0.4 and a LOD lather than 1.0, Goodness-of-fit for removal of loci = 5.0, Number of added loci after which to perform a ripple = 1, Third round = yes	

The Illumina PE reads were assembled by SOAPdenovo2 r223 (RRID: SCR_014986) [12] with *k*-mers of 61 and 81, and contigs were generated with total lengths of 352.2 Mbp (*k*-mer = 81) and 389.3 Mbp (*k*-mer = 61) (Table 2). The contigs constructed with *k*-mer = 81 were selected and scaffolded with mate-pair (MP) reads with insert sizes of 2, 5, 10, and 15 Kbp (kilobase pairs) by using SSPACE v2.0 (RRID: SCR_005056) [13]. The number of generated scaffolds was 6227 after gap filling by GapFiller [14] and excluding contamination. The total length of the scaffolds was 297.1 Mbp (Assembly 1, Table 2), which was approximately 55–92 Mbp shorter in length than the estimated genome size of HPK-4.

**Table 2:** Statistics of de novo whole genome assembly.

File name	SOAPdenovo Contigs		Assembly 1 SOAPdenovo/SSPACE/GapFiller (*k*-mer = 81)		Assembly 2 Platanus	Assembly 3 MaSuRCA	Assembly 4 TruSPAdes	Assembly 5 GMcloser	MUN_r1.1	MUN_r1.11	MUN_r1.11 pseudomolecule
Input reads	PE	PE	PE + MP	PE + MP	PE + MP	PE + MP	SLR	Assembly 1 + Assembly 4	Assembly 5	MUN_r1.1	MUN_r1.11 + Linkage map
Comments	*k*-mer = 61	*k*-mer = 81	Include contamination	Exclude contamination	Include contamination	Include contamination		Exclude contamination	≥500bp	Scaffolds revised	
All											

Number of sequences	1,534,576	779,101	7,123	6,228	62,323	17,400	374,253	6,228	3,495	3,497	10
Total length (bp)	389,388,347	352,263,669	297,816,217	297,127,168	287,695,252	313,146,882	1,357,659,302	295,740,202	294,688,765	294,688,765	259,245,825
Average length (bp)	254	452	41,811	47,708	4,616	17,997	3,628	47,486	84,317	84,269	25,924,583
Max length (bp)	43,864	86,786	13,495,995	13,495,995	13,114,378	9,397,721	79,948	13,482,853	9,844,273	9,844,273	33,386,276
Min length (bp)	62	82	300	300	100	71	1,500	146	500	500	15,505,026
N50 length (bp)	2,602	6,108	3,571,813	3,571,813	4,221,442	2,147,735	4,120	3,568,883	2,818,555	2,818,555	28,154,654
A	137,634,352	123,511,855	98,936,270	98,713,635	95,878,438	103,574,375	440,896,819	100,065,275	99,718,915	99,718,915	88,615,763
T	128,359,494	116,788,629	98,000,497	97,829,343	96,128,274	103,392,204	440,199,341	99,104,678	98,784,555	98,784,555	88,538,203
G	61,810,612	56,330,394	43,804,355	43,679,038	42,484,816	46,002,912	238,337,484	44,442,744	44,254,557	44,254,557	38,863,122
C	61,583,889	55,632,791	43,617,622	43,524,196	42,490,698	46,133,757	238,219,308	44,268,956	44,083,227	44,083,227	38,986,862
N	0	0	13,457,473	13,380,956	10,713,026	14,043,634	6,350	7,858,549	7,847,511	7,847,511	4,241,875
Total (ATGC, bp)	389,388,347	352,263,669	284,358,744	283,746,212	276,982,226	299,103,248	1,357,652,952	287,881,653	286,841,254	286,841,254	255,003,950
GC% (ATGC)	31.7	0	30.7	30.7	30.7	30.8	35.1	30.8	30.8	30.8	30.5
≥300 bp											
Number of sequences	96,834	85,229	7,123	6,228	13,045	17,107	374,253	6,226	-	-	-
Total length (bases)	255,385,256	270,010,759	297,816,217	297,127,168	281,104,166	313,093,559	1,357,659,302	295,739,758	-	-	-
Average length (bases)	2,637	3,168	41,811	47,708	21,549	18,302	3,628	47,501	-	-	-
≥500 bp											
Number of sequences	72,097	56,065	3,945	3,468	8,514	13,654	374,253	3,469	3,495	3,497	
Total length (bases)	245,981,429	258,994,765	296,598,533	296,074,149	279,298,951	311,725,703	1,357,659,302	294,688,765	294,688,765	294,688,765	
Average length (bases)	3,412	4,619	75,183	85,373	32,805	22,830	3,628	84,949	84,317	84,269	
≥1 Kbp											
Number of sequences	52,710	39,176	1,976	1,842	2,716	9,787	374,253	1,836	1,862	1,864	
Total length (bases)	232,331,716	247,369,100	295,266,774	294,976,239	275,198,779	308,936,166	1,357,659,302	293,585,247	293,585,247	293,585,247	
Average length (bases)	4,408	6,314	149,427	160,139	101,325	31,566	3,628	159,905	157,672	157,503	
≥2 Kbp											
Number of sequences	27,007	23,688	1,205	1,186	511	4,079	190,853	1,176	1,202	1,204	
Total length (bases)	185,671,228	219,707,018	294,019,523	293,893,601	272,047,316	299,058,101	960,490,176	292,496,469	292,496,469	292,496,469	
Average length (bases)	6,875	9,275	244,000	247,802	532,382	73,317	5,033	248,721	243,341	242,937	
≥3 Kbp											
Number of sequences	15,668	16,505	1,084	1,073	395	3,261	70,396	1,056	1,082	1,084	
Total length (bases)	141,491,218	191,540,893	293,553,905	293,455,161	271,611,649	295,920,059	495,770,522	292,032,037	292,032,037	292,032,037	
Average length (bases)	9,031	11,605	270,806	273,490	687,624	90,745	7,043	276,545	269,900	269,402	

We speculated that the shorter observed length of the total scaffolds may have been caused by misintegration of repeat sequences by SSPACE v2.0. Therefore, we performed subsequent assemblies using two programs, Platanus v1.2.1 (RRID: SCR_015531) [15] and MaSuRCA v2.3.2 (RRID: SCR_010691) [16]. The total ATGC lengths of the scaffolds were not significantly different among the three assemblies: 284.4 Mbp in SOAPdenovo-SSPACE (Assembly 1, before excluding contamination; Table 2), 277.0 Mbp in Platanus (Assembly 2), and 299.1 Mbp in MaSuRCA (Assembly 3).

Meanwhile, an Illumina synthetic long-reads (SLR) library was constructed with high-molecular-weight cellular DNA using a TruSeq synthetic long-read DNA library prep kit (Illumina). Sequences were generated by Illumina HiSeq 2000 and MiSeq systems with read lengths of 93 nt and 251 nt, respectively. The SLR reads were synthesized through the TruSPAdes v3.6.2 pipeline [17]. Among the three assemblies with PE and MP reads, Assembly 1 was used for subsequent analysis, and gaps were closed with Illumina SLRs by GMcloser (RRID: SCR_000646) [18]. Potentially contaminated sequences were excluded using BLASTN searches against the chloroplast and mitochondrial genome sequences of *Arabidopsis thaliana* (accession numbers NC_000932.1 and NC_001284.2), human genome sequences (hg19 [19]), fungal genome sequences registered with the National Center for Biotechnology Information (NCBI) [20], bacterial genome sequences registered with [21], vector sequences in UniVec [22], and PhiX (NC_001422.1) [23] sequences with *E*-value cutoffs of 1 × 10^−10^ and length coverage >10%. The total length of the resultant assembly (Assembly 5) was 295.7 Mbp.

The results of benchmarking universal single-copy ortholog (BUSCO) analysis (RRID: SCR_015008) [24] identified that 93.1% of BUSCOs were found as complete genes in Assembly 5. We therefore considered that Assembly 5 covered most of the coding regions of the horsegram genome. Sequences shorter than 500 bp were excluded from Assembly 5, and the remaining sequences were designated as MUN_r1.1.

### Linkage map and pseudomolecule construction

To construct chromosome-scale genome sequences, a SNP linkage map was created with the 214 F_2_ progenies. SNPs segregating in the F_2_ population were detected by mapping Illumina re-sequence reads of the eight F_2_ individuals onto the assembled genome using Bowtie2 (RRID: SCR_016368) [25], and by calling variants using SAMtools 0.1.19 (RRID: SCR_002105) [26] and vcftools 0.1.12 (RRID: SCR_001235) [27]. Target amplicon sequencing (TAS) was performed to genotype the identified SNPs according to the methods described in Shirasawa *et al.* [28].

The linkage map was constructed using JoinMap 4 with Kosambi’s mapping function (RRID: SCR_009248) [29]. The assembled genome sequence scaffolds were aligned onto the linkage map for pseudomolecule construction. The female parent of the F_2_ progenies was HPK-4. The male parent was initially considered to be HPKM-193, but this assignment was later found to be wrong when the whole genome sequences of HPK-4, HPKM-193, and the eight F_2_ progenies were compared. Candidate SNPs segregating in the F_2_ progenies were identified by mapping the whole genome Illumina sequences of the eight randomly selected F_2_ progenies onto MUN_r1.1.

**Figure 2.**
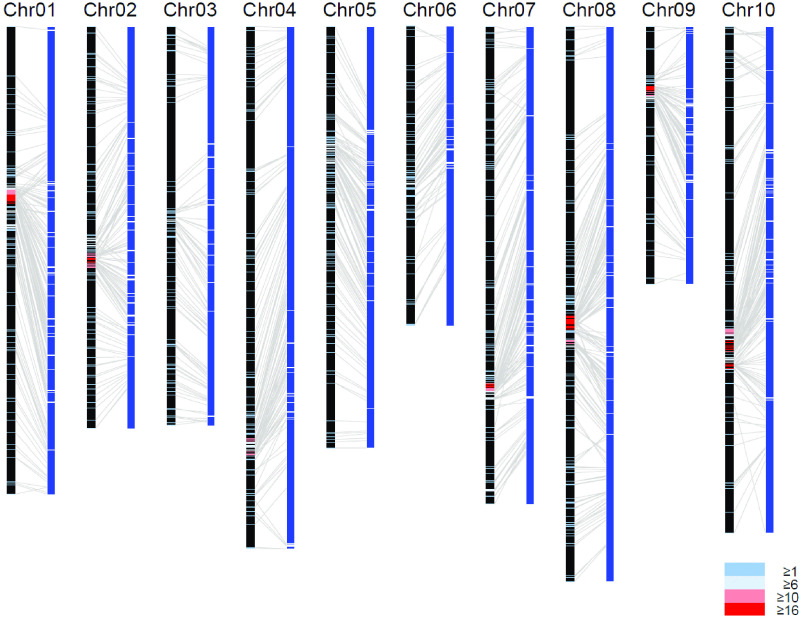
Anchoring the horsegram genome assembly to the genetic linkage map. The linkage groups (left black bars) and 219 anchored MUN_r1.1 scaffolds (right blue bars) with 1263 SNPs. The crossbars on the linkage groups show the positions of mapped SNPs. Blue, aqua, pink, and red colors represent the numbers of mapped SNPs per cM of 1–5, 6–10, 10–15, and ≧16, respectively.

A total of 2942 SNPs were identified, and 1378 SNPs were successfully genotyped by TAS analysis in 214 F_2_ progenies. Of these, 1263 SNPs were mapped onto the ten linkage groups with a total length of 980 cM (Table 3). A total of 219 scaffolds in MUN_r1.1 were then aligned onto the linkage map (Figure 2; Table 3; and in GigaDB [10]). During the process of alignment, two scaffolds were discovered to be misscaffoldings and split. The revised set of scaffolds was designated as MUN_r1.11 (Table 4; Table 2). The number of sequences of MUN_r1.11 was 3,495, with a total length of 294.7 Mbp and an N50 length of 2.8 Mbp. The aligned scaffolds on the linkage map were connected to 10,000 Ns for the construction of chromosome-scale pseudomolecules. The total length of the ten pseudomolecules was 259.2 Mbp, with an N50 length of 28.2 Mbp (Table 4; Table 5). When the total length of the A, G, T, and C bases was compared, the 10 pseudomolecules were found to cover 89% of the scaffolds in MUN_r1.11. The ratios of complete BUSCOs identified in MUN_r1.11 and the 10 scaffolds were 93.1% and 87.4%, respectively. Most of the complete BUSCOs were identified as single copies, suggesting a slow rate of duplication in the coding regions of the assembled genomes.

**Table 3:** Statistics of a SNP linkage map and numbers of anchored scaffolds.

	Linkage map				Number of anchored scaffolds (MUN_r1.1)
	Number of mapped SNPs	Length (cM)	Mean distance between SNPs (cM)	Segregation distortion ratio (%)	
Chr1	148	97.2	0.66	4.05	29
Chr2	128	73.1	0.57	74.22	26
Chr3	84	120.3	1.43	3.57	14
Chr4	131	123.1	0.94	14.50	18
Chr5	124	100.8	0.81	3.23	22
Chr6	76	88.9	1.17	14.57	16
Chr7	148	119.1	0.80	12.84	22
Chr8	185	117.1	0.63	2.70	19
Chr9	87	51.7	0.59	81.61	25
Chr10	152	88.8	0.58	3.95	28
Total	1,263	980	0.78		219

**Table 4:** Statistics on the horsegram genome assembly and CDS.

	MUN_r1.11	MUN_r1.11	MUN_r1.1_cds
	Genome/Scaffolds	Genome/Pseudomolecules	CDS
Number of sequences	3,497	10	36,105
Total length (bp)	294,688,765	259,245,825	38,820,013
Average length (bp)	84,269	25,924,583	1,075
Maximum length (bp)	9,844,273	33,386,276	15,732
Minimum length (bp)	500	15,505,026	150
N50 length (bp)	2,818,555	28,154,654	1,488
Total length of AGTC (bp)	286,841,254	255,003,950	
Gaps (bp)	7,847,511	4,241,875	-
GC%	30.8	30.5	43.8
Repeat %	28.99497136	-	-
Number of complete genes	-	-	35,508
Number of partial genes	-	-	597

**Table 5:** Assembly statistics of MUN_r1.11 pseudomolecules.

	MUN_chr01	MUN_chr02	MUN_chr03	MUN_chr04	MUN_chr05	MUN_chr06	MUN_chr07	MUN_chr08	MUN_chr09	MUN_chr10
Total length of sequences (bp)	28,154,654	24,194,727	23,973,329	31,423,847	25,354,798	18,013,159	28,753,260	33,386,276	15,505,026	30,486,749
A	9,658,539	8,292,913	8,231,116	10,813,335	8,677,934	6,151,137	9,805,582	11,440,621	5,247,870	10,296,716
T	9,689,700	8,302,615	8,197,375	10,734,237	8,667,169	6,161,839	9,816,935	11,432,900	5,212,370	10,323,063
G	4,127,487	3,524,028	3,621,541	4,739,623	3,794,983	2,702,465	4,354,257	5,051,796	2,292,810	4,654,132
C	4,128,504	3,558,607	3,614,862	4,779,430	3,830,211	2,738,375	4,343,032	5,030,174	2,307,484	4,656,183
N	550,424	516,564	308,435	357,222	384,501	259,343	433,454	430,785	444,492	556,655
Total (ATGC)	27,604,230	23,678,163	23,664,894	31,066,625	24,970,297	17,753,816	28,319,806	32,955,491	15,060,534	29,930,094
GC% (ATGC)	29.9	29.9	30.6	30.6	30.5	30.6	30.7	30.6	30.5	31.1
Number of anchored scaffolds	29	26	14	18	22	16	22	19	25	28
Total length of scaffolds	27,874,654	23,944,727	23,843,329	31,253,847	25,144,798	17,863,159	28,543,260	33,206,276	15,265,026	30,216,749
Total length of inserted Ns (N10000)	280,000	250,000	130,000	170,000	210,000	150,000	210,000	180,000	240,000	270,000

### Repetitive sequences

Repetitive sequences in the assembled genome were identified by RepeatMasker v3.2.9 (RRID: SCR_012954) [30] for known repetitive sequences registered in Repbase (RRID: SCR_021169) [31], and *de novo* repetitive sequences were defined by RepeatScout v1.0.5 (RRID: SCR_014653) [32]. A total of 50.2 Mbp of repetitive sequences were identified on the assembled genome, occupying 29% of the total length (Table 6). Of the identified repetitive sequences, the sequences registered in Repbase were found on 12.0% of the assembled genome, whereas unique repetitive sequences, i.e., those not registered in Repbase, were located on 17.0% of the assembled genome. Simple sequence repeat (SSR) motifs were identified by MISA mode in SciRoKo software with the default parameters (RRID: SCR_000941) [33]. A total of 74,362 SSRs were identified in MUN_r1.11 with an average frequency of 0.21 SSR per 100 Kbp [10]. The highest SSR frequency, 0.66 SSR per 100 Kbp, was observed in chr06, and this value was almost three times higher than that in chr03 and chr08 (0.22 SSR per 100 Kbp).

**Table 6:** Length and ratio of repetitive sequences.

					**MUN_r1.11**		**MUN_chr01**		**MUN_chr02**		**MUN_chr03**		**MUN_chr04**		**MUN_chr05**		**MUN_chr06**		**MUN_chr07**		**TSUd_chr08**		**MUN_chr09**		**MUN_chr10**	
					Length occupied (bp)	% of Whole genome^*b*) ^	Length occupied (bp)	% of Whole line-specific genomeb)	Length occupied (bp)	% of Whole line-specific genomeb)	Length occupied (bp)	% of Whole line-specific genomeb)	Length occupied (bp)	% of Whole line-specific genomeb)	Length occupied (bp)	% of Whole line-specific genomeb)	Length occupied (bp)	% of Whole line-specific genomeb)	Length occupied (bp)	% of Whole line-specific genomeb)	Length occupied (bp)	% of Whole line-specific genomeb)	Length occupied (bp)	% of Whole line-specific genomeb)	Length occupied (bp)	% of Whole line-specific genomeb)
Known repeats in Pepbase	Interspersed repeats	Class I	SINEs		33,194	0.0	1,893	0.0	2,704	0.0	2,330	0.0	3,998	0.0	3,017	0.0	2,162	0.0	5,597	0.0	3,606	0.0	769	0.0	3,640	0.0
			LINEs		758,450	0.3	79,135	0.3	60,158	0.2	78,223	0.3	67,284	0.2	61,531	0.2	35,312	0.2	76,948	0.3	95,328	0.3	32,464	0.2	88,614	0.3
			LTR elements	Total	18,946,454	6.4	2,502,167	8.9	1,905,724	7.9	1,430,860	6.0	1,626,139	5.2	1,474,156	5.8	938,839	5.2	1,864,330	6.5	2,080,923	6.2	1,268,235	8.2	1,796,168	5.9
				Copia	12,243,368	4.2	1,611,136	5.7	1,257,513	5.2	902,900	3.8	1,167,096	3.7	949,455	3.7	658,107	3.7	1,176,391	4.1	1,417,106	4.2	740,132	4.8	1,173,567	3.8
				Gypsy	6,273,336	2.1	820,553	2.9	601,000	2.5	500,201	2.1	417,277	1.3	496,833	2.0	254,838	1.4	654,972	2.3	613,122	1.8	491,823	3.2	594,170	1.9
		Class II	DNA elements		3,100,519	1.1	380,164	1.4	280,403	1.2	227,524	0.9	294,636	0.9	206,575	0.8	121,303	0.7	294,742	1.0	307,336	0.9	198,994	1.3	289,814	1.0
		Unclassified			420	0.0	0	0.0	77	0.0	0	0.0	0	0.0	0	0.0	0	0.0	0	0.0	243	0.0	0	0.0	63	0.0
	Helitrons	182,492	0.1	24,503	0.1	51,573	0.2	15,763	0.1	8,828	0.0	12,915	0.1	16,031	0.1	8,133	0.0	17,183	0.1	8,945	0.1	13,311	0.0			
	Low complexity^*a*)^	3,615,499	1.2	222,186	0.8	208,479	0.9	203,104	0.8	238,959	0.8	194,268	0.8	134,128	0.7	269,659	0.9	257,639	0.8	119,470	0.8	256,405	0.8			
	Simple repeat	8,038,181	2.7	744,057	2.6	655,110	2.7	581,825	2.4	793,431	2.5	634,665	2.5	493,067	2.7	687,667	2.4	783,434	2.3	367,990	2.4	753,700	2.5			
	Unknown	13,270	0.0	366	0.0	993	0.0	642	0.0	1,053	0.0	520	0.0	1,094	0.0	1,491	0.0	884	0.0	859	0.0	2,820	0.0			
	Subtotal	35,247,906	12.0	4,044,950	14.4	3,229,413	13.3	2,581,448	10.8	3,088,969	9.8	2,627,752	10.4	1,775,449	9.9	3,250,692	11.3	3,614,610	10.8	2,038,420	13.1	3,241,129	10.6			
Unique repeats	Unknown	49,554,792	16.8	6,234,995	22.1	4,672,589	19.3	3,435,357	14.3	4,094,114	13.0	3,347,931	13.2	2,243,739	12.5	4,226,300	14.7	5,021,333	15.0	3,433,883	22.1	5,105,098	16.7			
	Simple repeat	642,225	0.2	67,981	0.2	58,761	0.2	47,815	0.2	66,279	0.2	51,673	0.2	40,270	0.2	61,328	0.2	65,386	0.2	31,004	0.2	61,449	0.2			
	Subtotal	50,197,017	17.0	6,302,976	22.4	4,731,350	19.6	3,483,172	14.5	4,160,393	13.2	3,399,604	13.4	2,284,009	12.7	4,287,628	14.9	5,086,719	15.2	3,464,887	22.3	5,166,547	16.9			
Total					85,444,923	29.0	10,347,926	36.8	7,960,763	32.9	6,064,620	25.3	7,249,362	23.1	6,027,356	23.8	4,059,458	22.5	7,538,320	26.2	8,701,329	26.1	5,503,307	35.5	8,407,676	27.6

^*a*^Primarily poly-purine/poly-pyrimidine stretches, or regions of extremely high AT or GC content. Stretches of DNA (100 bp) were masked when they were >87% AT or >89% GC, and 30 bp stretches were masked when they contained 29 A/T (or GC) nucleotides.^*b*^N bases were excluded from the calculation.

### Transcript sequencing, gene prediction, and annotation

**Figure 3.**
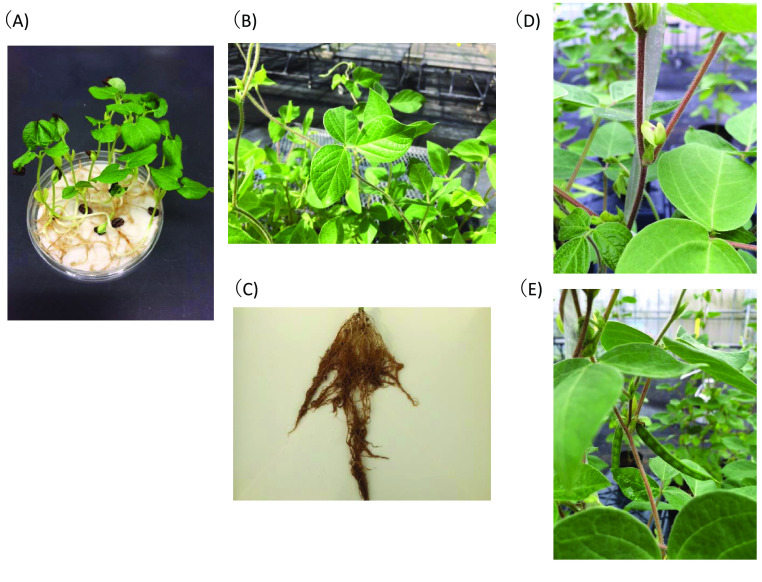
Plant materials used for transcript sequences. The seedlings (A), leaves (B), roots (C), flowers (D), and young pods (E) of HPK-4 used for Illumina transcript sequencing.

Total RNA of HPK-4 was extracted from seedlings, leaves, roots, flowers, and young pods using the RNeasy Plant Mini Kit (QIAGEN). RNA libraries were constructed by using a TruSeq standard mRNA HT sample prep kit (Illumina). Library sequencing was performed by an Illumina HiSeq system with a read length of 93 nt. Assembly was performed by Trinity [34]. A total of 485 million transcript Illumina reads were obtained from seedlings, leaves, roots, flowers, and young pods of HPK-4 (Figure 3) [10].

*Ab initio* gene prediction was performed by BRAKER1 v1.9 (RRID: SCR_018964) [35] with the obtained transcript sequences. Transposable elements (TEs) were detected by BLASTP searches against the NCBI NR protein database [36] with an *E*-value cutoff of 1 × 10^−10^. Domain search was performed by InterProScan against the InterPro database with an *E*-value cutoff of 1.0 (RRID: SCR_005829) [37].

A total of 46,095 gene sequences were predicted on the assembled genome with a total length of 48.3 Mbp (Table 7). After removal of TEs and both pseudo and short gene sequences, 36,105 gene sequences remained, and this set of sequences was designated as MUN_r1.1_cds (Table 4). The ratio of complete BUSCOs identified on MUN_r1.1_cds was 91.2%. Of the 36,105 sequences, 35,508 were classified as complete genes and 597 as partial. The coding sequences (CDSs) were further tagged with “f” (full similarity), “p” (partial similarity), and “d” (domain) according to the similarity level against the non-redundant database (f: *E*-values ≤1 × 10^−20^ and identity ≥70%; p: *E*-values ≤1 × 10^−20^ and identity <70%) and the InterPro database (d: *E*-values ≤1.0; Table 8). Of the 36,105 sequences, 21,471 (59.4%) were tagged with “f” and 6,692 (18.5%) with “p”. The number of gene sequences tagged with “d” was 24,575 (68.1%).

**Table 7:** Statistics of candidate genes predicted by BRAKER1 v1.9.

	All predicted genes	MUN_r1.1_cds
		Exclude TE, pseudo and short genes
Number of sequences	46,095	36,105
Total length (bp)	48,277,179	38,820,013
Average length (bp)	1,047	1,075
Max length (bp)	15,732	15,732
Min length (bp)	60	150
N50 length (bp)	1,443	1,488
GC%	43.3	43.8

**Table 8:** Number of CDSs showing significant similarity by BLASTP and domain searches against NCBI NR and InterPro.

Number of CDSs	% to Total		Classification	Tag		All predicted genes	MUN_r1.1_cds
	All predicted genes	MIN_r1.1_cds		Similarity against NR	Domain		
19,874	43	55	Complete	f	d	Included	Included
1554	3	4	Complete	f	-	Included	Included
3574	8	10	Complete	p	d	Included	Included
2996	6	8	Complete	p	-	Included	Included
1052	2	3	Complete	-	d	Included	Included
6458	14	18	Complete	-	-	Included	Included
26	0	0	Partial	f	d	Included	Included
17	0	0	Partial	f	-	Included	Included
49	0	0	Partial	p	d	Included	Included
73	0	0	Partial	p	-	Included	Included
124	0	0	Partial	-	d	Included	Included
308	1	1	Partial	-	-	Included	Included
126	0.3	-	Pseudo			Included	Not included
107	0.2	-	Short			Included	Not included
9757	21.2	-	TE			Included	Not included

BLASTP Search against NCBI NR database f: *E*-value ≦1 × 10^−20^ and similarity ≧70% p: *E*-value ≦1 × 10^−20^ and similarity <70% InterProScan against the InterPro database d: *E*-value ≦1.0

 Transfer RNA genes were predicted using tRNAscan-SE ver. 1.23 with the default parameters [38], and compared with the numbers on the genomes of *Phaseolus vulgaris* (Pvulgaris_218_v1.0, 681) [39], *Vigna angularis* (Vangularis_v1.a1) [40], *Lotus japonicus* (Lj3.0) [41], and *A. thaliana* (Araport11 [42]). Total number of putative tRNA genes in the assembled genomes (MUN_r1.11) was 690, almost the same as the numbers for the genomes of *P. vulgaris* (681), *V. angularis* (667), and *A. thaliana* (699, Table 9). rRNA genes were predicted by BLAST searches (*E*-value cutoff of 1 × 10^−10^) with query sequences of *A. thaliana* 5.8S and 25S rRNAs (X52320.1) and 18S rRNA (X16077.1). The total number of putative rRNA genes identified in the genome was 139, which was again the same as the number in the *P. vulgaris* genome.

**Table 9:** Numbers of putative tRNA and rRNA encoding genes identified in MUN_r1.1 and other legume species.

tRNA					
Encode	*M. uniflorum* (MUN_r1.11)	*P. vulgaris* (Pvulgaris_218_v1.0)	*V. angularis* (Vangularis_v1.genome)	*L. japonicus* (Lj3.0_pseudomol)	*A. thaliana* (TAIR10_genome)
Ala	40	41	44	40	33
Arg	34	43	52	54	39
Asn	17	22	30	28	19
Asp	27	30	32	32	28
Cys	12	16	20	76	17
Gln	21	21	24	23	19
Glu	31	28	32	40	27
Gly	46	51	44	48	43
His	13	15	14	19	12
Ile	101	30	32	29	25
Leu	49	56	53	57	45
Lys	34	43	35	41	33
Met	38	39	43	48	31
Phe	21	28	20	22	17
Pro	43	48	36	46	68
Ser	50	50	51	44	72
Thr	690	28	24	36	26
Trp	15	16	17	21	16
Tyr	15	17	18	22	83
Val	36	41	37	34	32
Subtotal	669	663	658	760	685
Subtotal (%)	97.0	97.4	98.7	88.6	98.0
Pseudo	19	12	7	80	13
SeC	0	1	0	12	0
Sup	0	1	0	0	0
Undet	2	4	2	6	1
Total	690	681	667	858	699
**rRNA**					
**Encode gene**	***M. uniflorum* (MUN_r1.1)**	***P. vulgaris* (Pvulgaris_218_v1.0)**	***V. angularis* (Vangularis_v1.genome)**	***L. japonicus* (Lj3.0_pseudomol)**	***A. thaliana* (TAIR10_genome)**
18S	40	48	224	27	4
25S	87	83	421	64	5
5.8S	12	8	139	6	2
Total	139	139	784	97	11

^*a*^Pseudo, SeC, Sup, and Undet represent pseudogenes, selenocysteine tRNAs, possible suppressor tRNAs, and tRNAs with undetermined/unknown isotypes, respectively.^*b*^Accession numbers used for identification or 5.8S, 18S, and 25S rRNAs were X52320.1, X16077.1, and X52320.1, respectively.

### Diversity analysis in genetic resources

**Figure 4.**
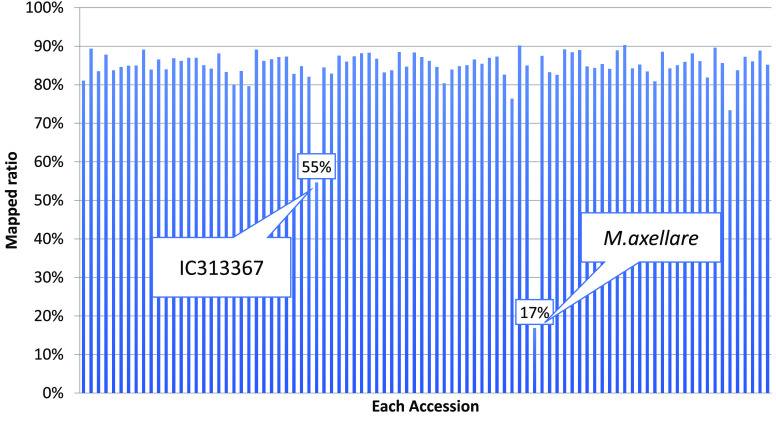
Mapped ratios of the dd-RAD-Seq reads of 92 accessions.

Only two species in the genus *Macrotyloma*, i.e., horsegram and *M. geocarpum*, are used as crops. It was speculated that horsegram domestication occurred in India twice: once in northwestern India at 4000 years before present, and once on the Indian Peninsula at 3500 years before present [43]. In addition, horsegram has narrow genetic diversity, as revealed by molecular analysis [44]. The genetic diversity of 91 cultivated horsegram accessions and one *M. axellare* accession, a wild relative of horsegram that is maintained at CSK-HPAU, were investigated based on dd-RAD-Seq analysis [10]. Library construction and variant calling were performed according to Shirasawa *et al.* [45]. The ddRAD-Seq reads were generated by an Illumina HiSeq 2000 system with a read length of 93 nt and mapped onto the assembled genome sequences. The two accessions, IC139449 and IC547543, were excluded from further analysis because of the small number of obtained reads. The mapped ratio of the reads onto the genome (MUN_r1.11) ranged from 80–90% in most of the accessions (Figure 4). However, *M. axellare* and one horsegram accession (IC313367) showed low mapping ratios of 17% and 55%, respectively. *M. axellare* was excluded from further analysis because of its low mapping ratio.

**Figure 5.**
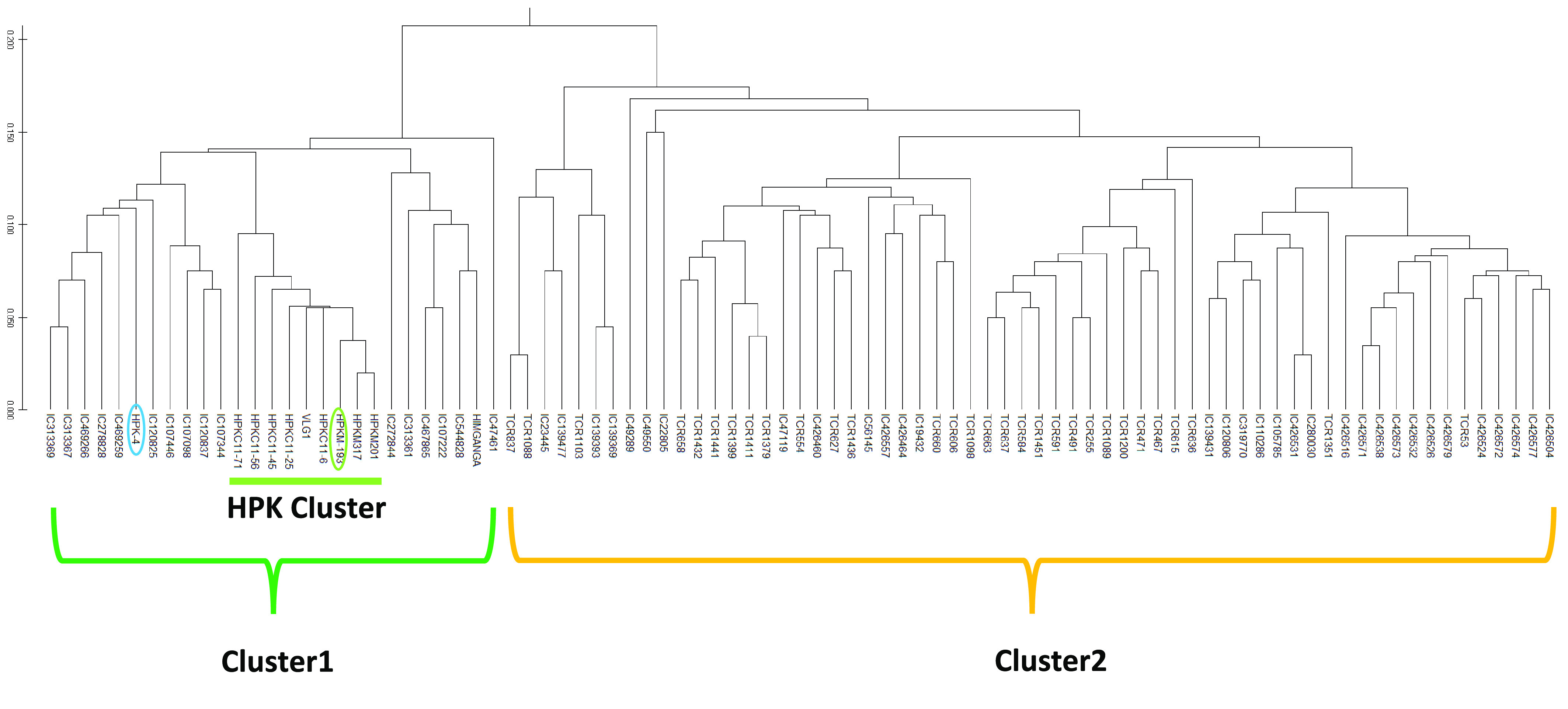
A phylogenetic tree of the 89 horsegram accessions based on 277 SNPs. HPK-4, used in the reference genome construction; HPK-4, used in the reference genome construction, and HPKM-193; the obtained whole genome sequences of the accessions are circled with blue and green lines, respectively.

 A total of 277 SNPs were identified in the remaining 89 accessions across the genome [10]. The Jaccard similarity coefficients of the 277 SNPs were calculated using GGT 2.0 [46], and a neighbor-joining (NJ) phylogenetic tree was constructed using MEGA ver 10.1.8 (RRID: SCR_000667) [47]. The NJ tree classified the 89 accessions into two clusters (Figure 5). Cluster 1 included varieties bred in the CSK-HPAU, which are prefixed with “HPK”. Most of the HPK varieties showed very close genetic relations and formed a subcluster (HPK cluster); the single exception was HPK-4.

### Whole genome structure in horsegram

**Figure 6.**
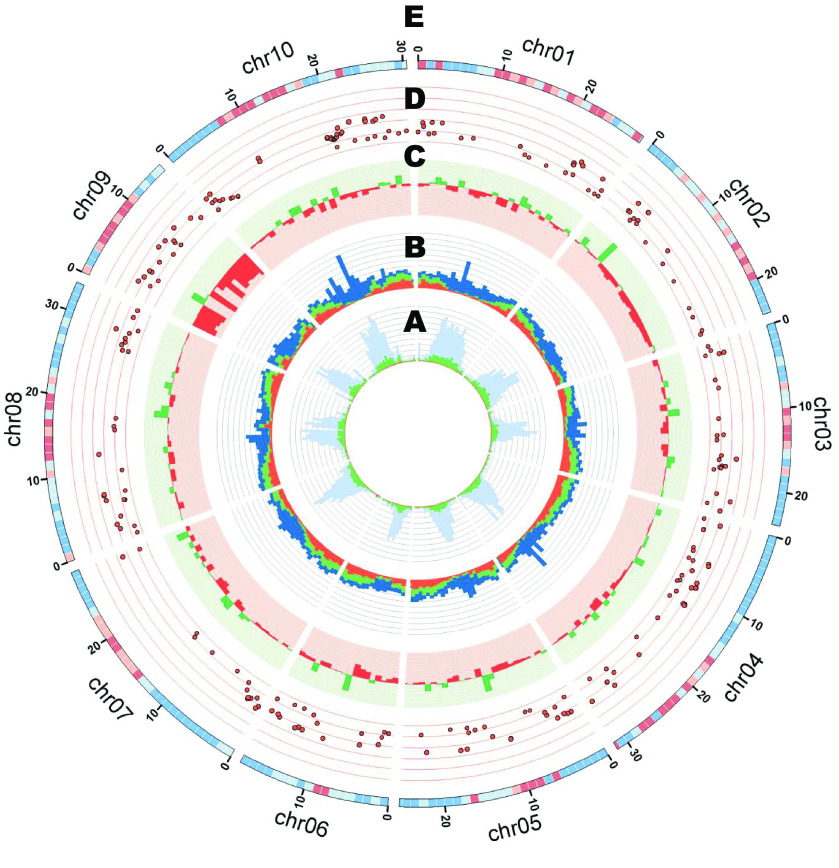
Graphical view of the horsegram genome structure. (**A**) Ratios of repetitive sequences in 1-Mbp windows. Blue bars represent horsegram-specific sequences. Green bars show sequences commonly observed in horsegram and three other legume species: *P. vulgaris*, *V angularis*, and *L. japonicus*. Red bars show sequences commonly observed in the four legume species and *A. thaliana*. (**B**) Numbers of the predicted horsegram genes (MUN_r1.1_cds) in 1-Mbp windows. The bar colors are the same as in (A). (**C**) CNV distribution in a 1-Mbp window. Green and red dots show log2 ratio plus and minus values, respectively. (**D**) Pi values and positions of the 255 SNPs identified in the 89 horsegram accessions by using dd-RAD-Seq. The distance between horizontal lines represents a Pi value of 0.1. (**E**) SNP density identified among the F_2_ mapping population based on whole genome re-sequencing of the eight F_2_ progenies. Blue, pale blue, pale pink, and pink indicate numbers of SNPs ≦50, ≦100, ≦150, and 150 < in 1-Mbp windows, respectively.

Figure 6 shows a graphical view of the horsegram genome structure with a graph drawn by Circos (Figure 6; RRID: SCR_011798) [48]. Repetitive sequences were frequently observed in the midsection of each chromosome, and the tendency was more pronounced in horsegram-specific sequences (Figure 6A). The ratio of repetitive sequences commonly observed in all five species was quite low, suggesting the uniqueness of repetitive sequences compared to the gene sequences. The gene sequences commonly observed between horsegram and the other compared species tended to be distributed to the two end regions of the chromosomes (Figure 6B). On the other hand, horsegram-specific gene sequences were distributed more uniformly across the genome, suggesting the unique structure of the horsegram genome.

 Copy number variations (CNVs) of one horsegram accession, HPKM-193, were detected against the HPK-4 genome (Figure 6C) based on the whole genome sequence reads of HPKM-193 using CNV-Seq (RRID: SCR_013357) [49] with a 1-Mbp window. CNVs with a minus log2 ratio were particularly observed on chr09 and chr02.

Of the 277 SNPs identified among the 89 horsegram accessions, 255 were located across the genome sequences of 10 chromosomes (Figure 6D). In each chromosome, SNPs were mostly identified in the regions where common putative genes of horsegram and the other compared species were located, particularly for chr04, chr07, chr08, and chr10. The differing trends in variable distribution is thought to reflect the presence of varying degrees of selection pressure in the horsegram germplasm resources in Himachal Pradesh.

SNP density mapped on the linkage map is illustrated in Figure 6E. As in the case of the CNVs, distribution bias was observed in the SNPs of HPKM-193; however, this bias was not like that in CNVs. A higher SNP density was observed in the midsection in most of the chromosomes. Chr06 showed less variation than the other chromosomes.

### Genes related to drought tolerance

Horsegram is considered one of the most drought-tolerant legume crop species. Personal investigation showed that plants can survive for more than 20 days without water under controlled conditions. A study by Bhardwaj *et al.* [50] described a transcriptome analysis of eight shoot and root tissues of a drought-sensitive (M-191) genotype and a drought-tolerant (M-249) genotype of horsegram under controlled and drought stress conditions. This study identified some important genic regions responsible for drought tolerance.

To estimate genes related to drought tolerance in the horsegram genome, a BLASTP search of the 36,105 putative genes was performed against amino acid sequences of *A. thaliana* (Araport11), and hit genes were further used in BLAST searches against DroughtDB [51], the NCBI NR protein database, and Plant Stress Gene Database [52]. A total of 158 horsegram genes showed significant similarity to the 78 genes in DroughtDB [10]. The most frequently hit gene was ABCG40, which encodes a protein that functions as an ABC transporter, and showed significant similarity to 14 horsegram genes. OST1/SRK2E and AtrbohF were also frequently identified, with hits to seven and six horsegram genes, respectively. Of the 158 genes, 93 showed the same domain sequences as the *A. thaliana* gene, and 52 were like the genes registered in the PSGD. These genes were indicated to have a greater likelihood of being candidate genes related to drought tolerance.

### Comparative and phylogenetic analyses with other legume species

Horsegram belongs to the subtribe *Phaseolinae* in the millettioid clade, along with *P. vulgaris* and *V. angularis*. The genome structure of horsegram was compared with those of *P. vulgaris* (Pvulgaris_218_v1.0), *V. angularis* (Vangularis_v1.a1), and *L. japonicus* (Lr_r3.0).

**Figure 7.**
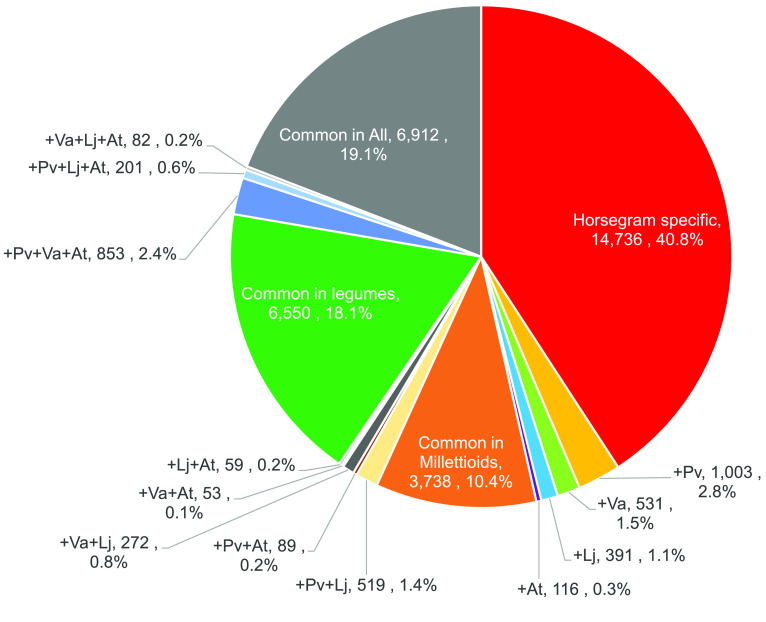
Ratios of genes of horsegram (MUN_r1.1_cds) clustered with those of four other plant species. Pv, Va, Li, and At represent genes of *P. vulgaris* (Pvulgaris_218_v1.0), *V. angularis* (Vangularis_v1.a1), *L. japonicus* (Lj_r3.0), and *A. thaliana* (Araport11), respectively.

The predicted gene sequences in MUN_r1.1_cds were clustered with other plant species (*P. vulgaris*, *V. angularis*, *L. japonicus*, and *A. thaliana*) for comparison at the protein sequence level. A total of 73,457 clusters were generated using the program CD-HIT (RRID: SCR_007105) [53] (Table 10). Of the 36,105 putative gene sequences, 21,369 (59.2%) genes were clustered with other plant species and 14,736 (40.8%) were considered horsegram-specific genes (Figure 7). A total of 3738 (10.4%) horsegram gene sequences were clustered with 3,864 *P. vulgaris* and 3,713 *V. angularis* genes, which were considered millettioid-specific genes. Common genes in legumes were identified for 6550 (18.1%) horsegram gene sequences, based on clusters with *P. vulgaris*, *V. angularis*, and*L. japonicus*.

**Table 10:** Number of gene clusters in horsegram and the four plant species, *P. vulgaris* (Pvulgaris_218_v1.0), *V. angularis* (Vangularis_v1.a1), *L. japonicus* (Lj_r3.0), and *A. thaliana* (Araport11).

Clustered species	Number of clustered species	Number of clusters	Number of clustered genes				
			Horsegram	*P. vulgaris* (Pv)	*V. angularis* (Va)	*L. japonicus* (Lj)	*A. thaliana* (At)
Horsegram	1	9,578	14,736	0	0	0	0
*P. vulgaris* (Pv)	1	3,449	0	4,114	0	0	0
V. angularis (Va)	1	8,306	0	0	9,545	0	0
*L. japonicus* (Lj)	1	17,544	0	0	0	21,677	0
*A. thaliana* (At)	1	15,177	0	0	0	0	18,129
Horsegram + Pv	2	897	1,003	1,028	0	0	0
Horsegram + Va	2	471	531	0	530	0	0
Horsegram + Lj	2	362	391	0	0	484	0
Horsegram + At	2	111	116	0	0	0	147
Pv + Va	2	1,031	0	1,248	1,159	0	0
Pv + Lj	2	262	0	293	0	333	0
Pv + At	2	88	0	97	0	0	130
Va + Lj	2	290	0	0	317	382	0
Va + At	2	92	0	0	95	0	113
Lj + At	2	326	0	0	0	398	415
Horsegram + Pv + Va (Common in Millettioids)	3	3,189	3,738	3,864	3,713	0	0
Horsegram + Pv + Lj	3	458	519	521	0	594	0
Horsegram + Pv + At	3	87	89	92	0	0	110
Horsegram + Va + Lj	3	230	272	0	255	292	0
Horsegram + Va + At	3	49	53	0	54	0	58
Horsegram + Lj + At	3	56	59	0	0	72	74
Pv + Va + Lj	3	581	0	649	664	725	0
Pv + Va + At	3	94	0	101	99	0	118
Pv + Lj + At	3	66	0	79	0	79	89
Va + Lj + At	3	41	0	0	45	51	53
Horsegram + Pv + Va + Lj (common in legumes)	4	5,011	6,550	6,591	6,472	6,847	0
Horsegram + Pv + Va + At	4	670	853	857	845	0	873
Horsegram + Pv + Lj + At	4	173	201	196	0	222	208
Horsegram + Va + Lj + At	4	76	82	0	83	102	98
Pv + Va + Lj + At	4	302	0	370	365	420	385
Common in all	5	4,390	6,912	7,097	7,000	7,056	6,638
Number of genes			36,105	27,197	31,241	39,734	27,621
Number of clustered genes			36,105	27,197	31,241	39,734	27,638
Number of non-clustered genes			0	0	0	0	17

**Figure 8.**
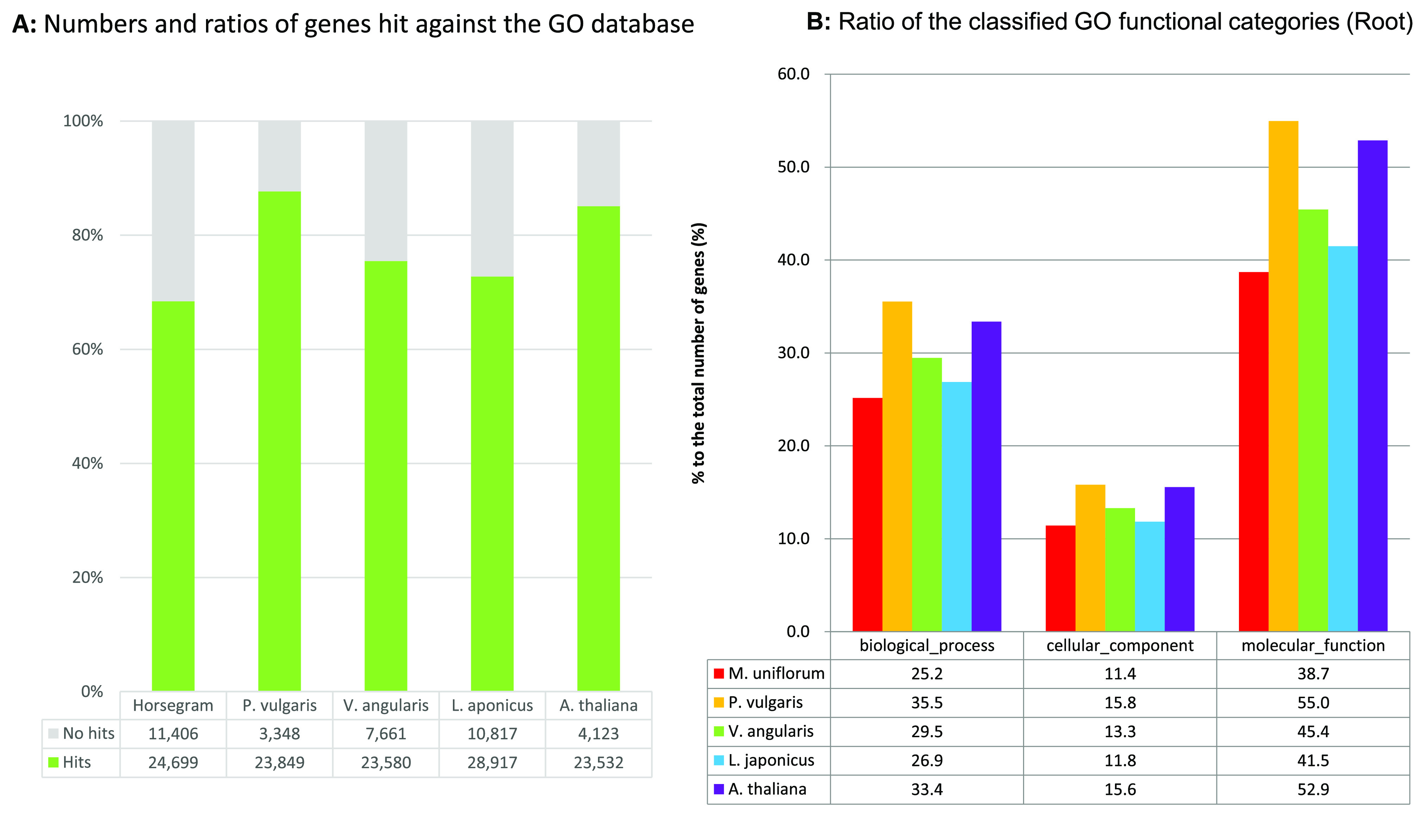
Comparison of genes annotated by the GO database in horsegram (MUN_r1.1_cds), *P. vulgaris* (Pvulgaris_218_v1.0), *V. angularis* (Vangularis_v1.a1), *L. japonicus* (Lj_r3.0), and *A. thaliana* (Araport11). (**A**) Numbers and ratios of genes annotated by GO database. (**B**) Ratios of the classified GO categories in the predicted genes.

**Figure 9.**
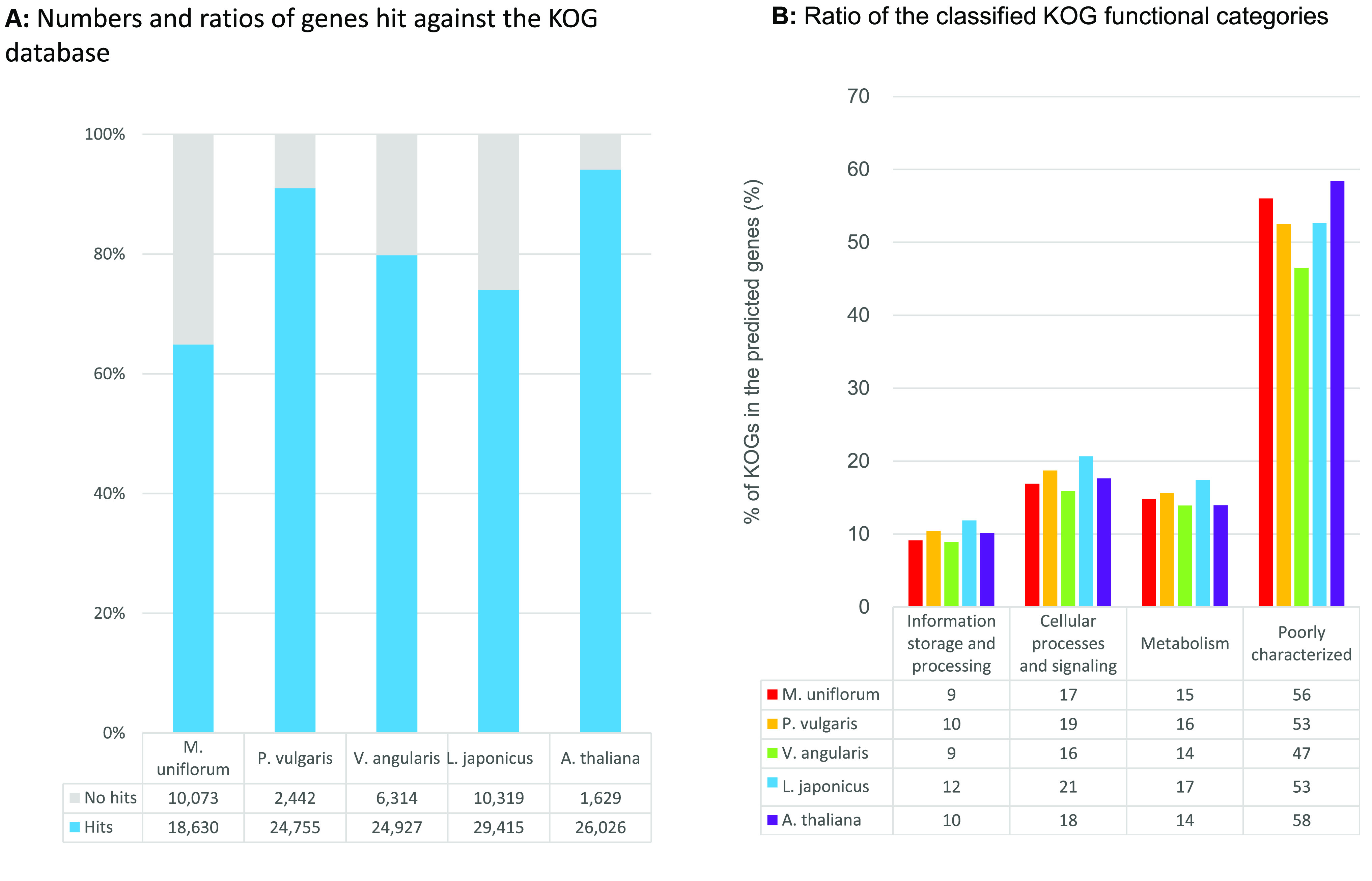
Comparison of genes annotated by the KOG database in horsegram (MUN_r1.1_cds), *P. vulgaris* (Pvulgaris_218_v1.0), *V. angularis* (Vangularis_v1.a1), *L. japonicus* (Lj_r3.0), and *A. thaliana* (Araport11). (**A**) Numbers and ratios of genes annotated by the KOG database. (**B**) Ratio of the classified KOG categories in hit genes.

Functional analysis was performed for MUN_r1.1_cds by classifying 36,105 putative genes into the Gene Ontology (GO) and euKaryotic clusters of Orthologous Groups (KOG) databases [54]. A total of 24,699 (68.4%) putative genes were annotated with GO categories including 9086 (25.2%) genes involved in biological processes, 4127 (11.4%) genes coding for cellular components, and 1377 (38.7%) genes associated with molecular functions (Figure 8). The ratio of annotated horsegram genes was smaller than those of the other species. The species with a ratio of classified GO categories most like that of horsegram was *L. japonicus*. A total of 18,630 (51.6%) putative genes showed significant similarity to genes in the KOG database (Figure 9). As in the results for GO, the ratio of hit genes was lower than for the other four species.

**Figure 10.**
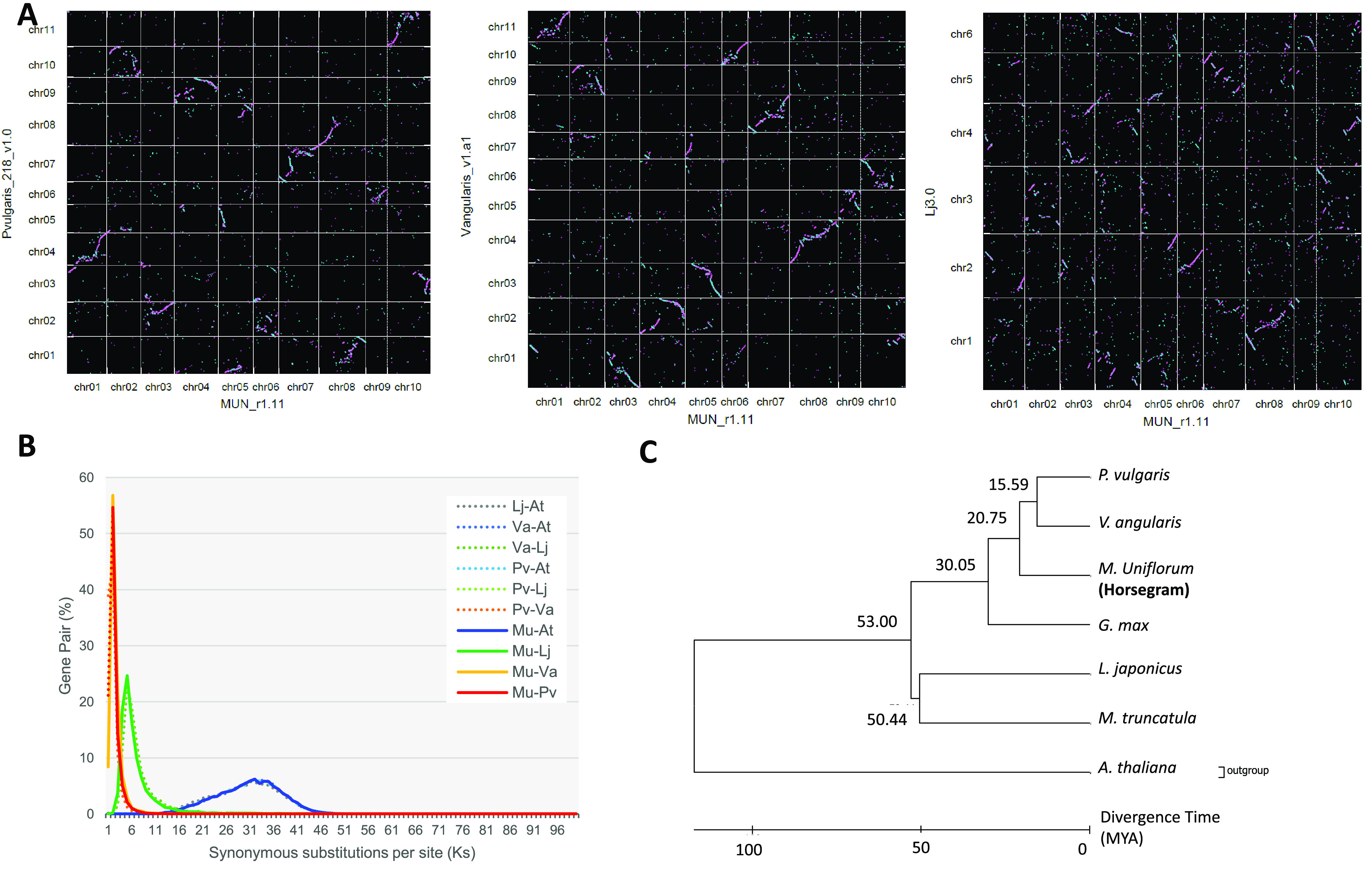
Comparative and phylogenetic analyses with other legume species. (**A**) Graphical view of syntenic relationships between horsegram and *P. vulgaris* (left), *V. anagularis* (middle), and *L. japonicus* (right). Pink and blue dots show homologous sequences of MUN_r1.11 with forward and reverse directions against the reference sequences. (**B**) Distribution of Ks values of orthologous gene pairs in horsegram (Mu) and the four plant species: *P. vulgaris* (Pv), *V. anagularis* (Va), *L. japonicus* (Lj), and *A. thaliana* (At). **C:** Phylogenetic tree of 4,154 common single-copy genes of the six legume species: *P. vulgaris*, *V. anagularis*, *G. max*, *L. japonicus*, *M. truncatula*, and *A. thaliana*.

 Clear relationships were observed with a warm-season legume, *V. angularis*, and one-on-one relationships were observed between horsegram chr02 (Mun_chr02) and *V. angularis* chr09 (Va_chr09), Mun_chr04 and Va_chr02, Mun_chr06 and Va_chr10, Mun_chr07 and Va_chr08, Mun_chr08 and Va_chr04, and Mun_chr09 and Va_chr05 (Figure 10A). The syntenic relations with *P. vulgaris* were slightly more complex than those with *V. angularis*, and those with the cool-season legume *L. japonicus* were more fragmented.

 Synonymous substitutions per site (Ks) were estimated by comparing gene pairs in each combination of horsegram, *P. vulgaris*, *V. angularis*, *L. japonicus*, and *A. thaliana* (Araport11) by KaKs Calculator [55] based on the clustered genes using the CD-HIT program (Figure 10B). The similar distributions of horsegram, *P. vulgaris*, and *V. angularis* indicated the close relations among the three species. The ratios of gene pairs showing Ks values less than 0.1% were 21.1% between horsegram and *P. vulgaris* and 8.4% between horsegram and *V. angularis*, suggesting that there was a closer relationship between horsegram and *P. vulgaris* at the gene level.

The phylogenetic analysis was performed with *Medica truncatula* (r5.0) [56] and *Glycine max* (Glma4 [57]) in addition to *P. vulgaris*, *V. angularis*, *L. japonicus*, and *A. thaliana*. A total of 978 common single-copy genes were identified for horsegram and the six species by clustering the genes using OrthoFinder (RRID: SCR_017118) [58]. Multiple alignment was performed for the 978 single-copy genes using Muscle (RRID: SCR_011812) [59], and gaps were excluded by Gblock [60]. An NJ tree was created with the 4154 single-copy orthologous genes identified in the four legume species by MEGA 7.0.9 beta (RRID: SCR_000667) [61] and TIMETREE (RRID: SCR_021162) [62]. *A. thaliana* was used for the outgroup (Figure 10C). When the divergence time between *M. truncatula* and *G. max* was considered to be 53 million years ago, it was estimated that horsegram diverged from *P. vulgaris* and *V. angularis* 20.75 million years ago (Figure 10C). Among the four legume species in millettioids, *P. vulgaris* and *V. angularis* shared closer relations with each other than with horsegram, and horsegram was closer to *P. vulgaris* and *V. angularis* than to *G. max*. The results are in consonance with a previous study based on a comparison of eight chloroplast regions [63].

### Data validation and quality control

The quality of assembled genome and gene sequences was investigated by using 1375 Embryophyta BUSCOs (v 3.0, obd10; RRID: SCR_015008) [24]. A total of 1340 (93.1%) BUSCOs were identified on the assembled scaffolds (MUN_r1.11), while 1259 (87.4%) and 1313 (91.2%) were converted by the pseudomolecules and CDS sequences (Table 11).

**Table 11:** Statistics of the horsegram genome assembly and CDS.

	MUN_r1.11	MUN_r1.11	MUN_r1.1_cds
	Genome/Scaffolds	Genome/Pseudomolecules	CDS
BUSCOs			
Complete	1340 (93.1%)	1259 (87.4%)	1313 (91.2%)
Complete single-copy	1252 (86.9%)	1181 (82%)	1208 (83.9%)
Complete duplicated	88 (6.1%)	78 (5.4%)	105 (7.3%)
Fragmented	26 (1.8%)	31 (2.2%)	23 (1.6%)
Missing	74 (5.1%)	150 (10.4%)	104 (7.2%)

### Reuse potential

In this study, we have provided a first-draft genome assembly of horsegram cultivar (HPK-4) and investigated features of the horsegram genome and gene sequences as well as the genetic diversity of the accessions. This information will help to establish an efficient breeding program for horsegram by integrating conventional breeding with marker-based biotechnological tools. Finally, the genomic information revealed in this study can be applied to the improvement of other disadvantageous food legumes.

## Data Availability

The genome assembly data, annotations, and gene models are available at the Horsegram Database [64]. The obtained genome sequence reads are available from the DNA Databank of Japan (DDBJ) Sequence Read Archive (DRA) under the BioProject accession number PRJDB5374. Data sets supporting the results of this article are available in *GigaScience* Database [10].

## List of abbreviations

BUSCO: benchmarking universal single-copy ortholog; CDS: coding sequence; CNV: copy number variation; CSK-HPAU: CSK Himachal Pradesh Agricultural University; GO: Gene Ontology; Kbp: kilobase pairs; KOG: euKaryotic clusters of Orthologous Groups; Mbp: megabase pairs; MP: mate-pair; NCBI: National Center for Biotechnology Information; NJ: neighbor-joining; PE: paired-end; SLR: synthetic long read; SNP: single nucleotide polymorphism; SSR: simple sequence repeat; TAS: target amplicon sequencing; TE: transposable element.
